# The Effect of* Pluchea indica* (L.) Less. Tea on Adipogenesis in 3T3-L1 Adipocytes and Lipase Activity

**DOI:** 10.1155/2018/4108787

**Published:** 2018-07-12

**Authors:** Kittipot Sirichaiwetchakoon, Gordon Matthew Lowe, Kanjana Thumanu, Griangsak Eumkeb

**Affiliations:** ^1^School of Preclinic, Institute of Science, Suranaree University of Technology, 111 University Avenue, Suranaree Subdistrict, Muang District, Nakhonratchasima 30000, Thailand; ^2^School of Pharmacy and Biomolecular Sciences, Liverpool John Moores Univerisity, James Parsons Building, Byrom Street, Liverpool, UK; ^3^Synchrotron Light Research Institute (Public Organization), Nakhon Ratchasima, 30000, Thailand

## Abstract

Obesity and hyperlipidemia are a major problem in the world.* Pluchea indica* (L.) Less. tea (PIT) is a beverage that has various indications. This study focused on the effect of the PIT on inhibiting adipogenesis of 3T3-L1 cells and pancreatic lipase enzyme activity. The viability of 3T3-L1 cells was not significantly decreased after exposure to 200 to 1000 *μ*g mL^−1^ PIT compared to controls (*p* > 0.05). The PIT at 750 to 1000 *μ*g mL^−1^ exhibited a significantly reduced lipid accumulation compared to the control (*p* < 0.05). The inhibitory effects of the PIT at 250 to 1000 *μ*g mL^−1^ on lipase activity were significantly increased compared to control (*p* < 0.05). The FTIR results showed that the integrated areas of lipids, proteins, nucleic acids, glycogen, and carbohydrates of the PIT-treated 3T3-L1 adipocytes were significantly lower than the untreated 3T3-L1 adipocytes (*p* < 0.05). These findings may indicate that the PIT is not only capable of inhibiting lipids and carbohydrate accumulation in adipocytes but also has a potential to inhibit pancreatic lipase activity. So, the PIT may be further developed to the novel lipid-lowering herbal supplement for the management of overweight or obesity.

## 1. Introduction

The global prevalence of obesity is increasing worldwide rapidly [1]. Obesity is caused by an imbalance of energy intake and expenditure [2]. The World Health Organization estimates that over 1.5 billion adults are overweight based on Body Mass Index (BMI) ≥ 25 and over 400 million of them are obese based on BMI ≥ 30 [1]. The consequences of an obese population are that they are more prone to develop major health problems such as type 2 diabetes, ischemic heart disease, stroke, and cancer [3, 4]. It is necessary to treat obese individuals by encouraging a reduced calorific intake, inhibiting pancreatic lipase and adipocyte differentiation, and stimulating energy expenditure by increasing physical activity, but also by regulating lipid metabolism and the surgical option such as laparoscopic adjustable gastric banding (LABG) in morbid obesity [2, 5, 6]. Reducing fat digestion and absorption can be effective in treating obesity [2]. Pancreatic lipase is an important enzyme that can hydrolyse dietary triacylglycerol to glycerol and fatty acids in the intestine [7]. Glycerol and fatty acids are regarded as the end products of lipid digestion in the gut; the inhibition of pancreatic lipase may be considered as a fat reducing absorption therapy [7, 8]; that is one mechanism of obesity treatment [9].

The 3T3-L1 cell line is a preadipose cell line which is developed from mouse cells. The 3T3-L1 preadipocyte cells will differentiate into adipocyte cells under an appropriate condition [10]. The inhibition of adipogenesis in 3T3-L1 adipocyte cells by Oil Red O staining method can be implied to attenuate hyperlipidemia and obesity [11].

Fourier transform infrared microspectroscopy (FTIR) is a technique which is used to obtain an infrared spectrum of absorption or emission with high sensitivity of different functional groups such as lipids, carbohydrates, proteins, and nucleic acids in biological structure. In addition, FTIR has also been used for analysis biomolecular changes in 3T3-L1 adipocytes [11].

Conventional antiobesity and antihyperlipidemic drugs have limited efficacies and critical adverse effects such as Orlistat, often associated with rebound weight gain after the cessation of drug and many patients cannot tolerate its gastrointestinal side effects [12]. Simvastatin, an antihyperlipidemic drug, can cause severe adverse event such as rhabdomyolysis [13]. At present, there is an increased demand for using plants in therapy instead of using synthetic drugs because it may have minor adverse effects and traditional medicinal plants are often cheaper and easily consumable [14].

The plant* Pluchea indica *(L.) Less. (*P. indica*) (family: Asteraceae) is a large evergreen shrub found abundantly in salt marshes. The plant is also known to be used in rheumatoid arthritis [16]. The plant has also been reported to possess diuretic effects. So far some chemical constituents have been isolated from different parts of the plant [17]. Two new thiophene derivatives and two pentacyclic triterpenes have been isolated from the root of this plant [17, 18]. The methanolic extract of* P. indica* leaves showed a reduction in blood glucose level in normal (35.12% and 36.01% for 200 and 400 mg kg^−1^, respectively) and streptozotocin-induced diabetic rats (36.10% and 41.87% for 200 and 400 mg kg^−1^, respectively) [19]. The methanol fraction of* P. indica* root extract has been reported to possess significant hepatoprotective properties [20]. In a separate study the extract displayed significant anti-inflammatory activity against glucose oxidase induced paw oedema* in vivo*, inhibited hydroxyl radical and lysis of erythrocytes induced by hydrogen peroxide, and significantly reduced serum enzyme levels (AST, ALT, LDH, and serum alkaline phosphatase), serum bilirubin content in acute liver injury, and total serum protein, albumin, and albumin/globulin ratio [21].

The purpose of this study was to investigate the inhibitory effect of the PIT on pancreatic lipase activity and adipogenesis. The biochemical profile in 3T3-L1 adipocytes was also investigated using the FTIR technique. The total phenolic and flavonoid contents in the PIT were also measured.

## 2. Materials and Methods

### 2.1. Plant Materials

Fresh herb of* Pluchea indica* (L.) was collected from Nakhon Ratchasima and Northeast region of Thailand. The plant specimen was authenticated by Dr. Paul J Grote. Identification was made in comparison with the voucher specimen (BKF 194428) and deposited at Forest Herbarium, National Park, Wildlife, and Plant Conservation Department, Ministry of Natural Resources and Environment, Thailand. This herb was washed thoroughly. The production process was performed by the Crystal Biotechnology Co., Ltd., and Suranaree University of Technology. PIT dry sample was added to boiling distilled water. The concentration of PIT sample was calculated from concentration of* Pluchea indica* (L.) dry weight in distilled water (*μ*g mL^−1^), further heated for 10 min, and then filtered through Whatman No.1 paper.

### 2.2. Chemicals and Reagents

3T3-L1 mouse embryonic fibroblasts and bovine calf serum were purchased from the American Type Culture Collection (ATCC, USA). Dulbecco's Modified Eagle's medium (DMEM) with high glucose, Penicillin, Streptomycin, N-2-hydroxyethylpiperazine-N-2-ethane sulfonic acid (HEPES), and 3-(4,5-Dimethylthiazol-2-yl)-2,5-diphenyltetrazolium bromide (MTT) was obtained from Gibco Invitrogen (Grand Island, NY). Bovine calf serum (BCS), fetal bovine serum (FBS), and Oil Red O were obtained from Hyclone (Logan, Utah). Insulin solution from bovine, 3-Isobutyl-1-methylxanthine (IBMX), lipase from porcine pancreas, 4-nitrophenyl dodecanoate (pNP), Orlistat, gallic acid, Folin–Ciocalteau reagent, and catechin were obtained from Sigma-Aldrich (St. Louis, USA). Dexamethasone (DEX) was obtained from G Bioscience (St. Louis, USA). Dimethyl sulfoxide (DMSO) was obtained from Carlo Erba Reagents S.r.l. (Chaussée du Vexin, Val de Reuil, USA). 4-*O*-caffeoylquinic acid (4-CQ), 5-*O*-caffeoylquinic acid (5-CQ), 3,4-*O*-dicaffeoylquinic acid (3,4-CQ), 3,5-*O*-dicaffeoylquinic acid (3,5-CQ), and 4,5-*O*-dicaffeoylquinic acid (4,5-CQ) were purchased from Chengdu Biopurify Phytochemicals Ltd., (Sichuan, China). Other reagents used were all analytical grade.

### 2.3. Cell Culture

The 3T3-L1 preadipocytes were seeded in a 6-well plate at a density of 5×10^5^ cells/well and cultured in DMEM with high glucose, added with 100 U mL^−1^ penicillin, 100 *μ*g mL^−1^ streptomycin, and 10% bovine calf serum until confluent. The cells were maintained at 37°C in 5% CO_2_ and 95% humidity.

### 2.4. Differentiation Procedures

Two days after confluence (day 0), the 3T3-L1 preadipocyte cells were induced to differentiate into adipocytes by adding differentiation medium containing 1.0 *μ*g mL^−1^ insulin, 1.0 *μ*M dexamethasone, 10% FBS, and 0.5 mM of IBMX in DMEM for 48 h (day 2). The differentiation medium was changed to maintain medium on day 2. The maintenance media consisted of 1.0 *μ*g mL^−1^ insulin and 10% FBS in DMEM for 48 h (day 4). The medium was replaced every 48 h until day 10. The 3T3-L1 preadipocytes were treated with various concentrations of the PIT at final concentrations (250 - 1000 *μ*g mL^−1^) for 48 h during periods of the differentiation phase (at day 0, 2, 4, 6, and 8). At day 10, the differentiation of 3T3-L1 preadipocytes was observed.

### 2.5. *In Vitro* Cytotoxic Test (MTT Assay)

The cytotoxic effect of the PIT on cell proliferation was determined using the MTT assay [22]. Briefly, the cells were seeded in a 96-well plate at a density of 5×10^3^ cells/well. The cells were allowed to adhere for 48 h and then were induced to differentiate into adipocytes by adding differentiation medium and treated with PIT between 250 and 1000 *μ*g mL^−1^. The differentiation medium was changed to maintain medium and treated with a PIT in various concentration on day 2, and the maintenance medium with a PIT in various concentrations was replaced every 48 h until day 10. At day 10, the cytotoxic effect of the PIT on cell proliferation was investigated. The culture medium was removed and 0.5 mg mL^−1^ MTT reagent was added, and the cells were incubated for 4 h at 37°C. The viable cells formed formazan crystal and were dissolved in DMSO. The absorbance was measured at 540 nm with a microplate spectrophotometer (Benchmark Plus, Bio-Rad, Japan).

### 2.6. Oil Red O and Hematoxylin Staining

Oil Red O staining can assess the increased amount of lipid accumulation normally associated with adipocyte differentiation [23]. Briefly, 3T3-L1 preadipocytes were induced to start adipogenesis by standard adipogenic medium and treated with a PIT at various concentrations (250, 500, 750, and 1000 *μ*g mL^−1^). After 48h, differentiation medium was changed to maintaining medium with different concentrations of the PIT. The medium with various concentrations of the PIT was replaced every 48 h with maintaining medium until day 10. The cells were washed with PBS twice and fixed with 10% formaldehyde in PBS for 1h. After that, cells were washed with distilled water twice and stained with 0.5% Oil Red O solution in 60:40 (v/v) isopropanol: distilled water for 30 min at room temperature. The Oil Red O stained cells were washed twice with distilled water and treated with hematoxylin solution for 10 min at room temperature. The triglyceride droplets were washed twice with 60% isopropanol, eluted with 100% isopropanol, and transferred to new 96 well plates. The lipid accumulation was quantified by measuring the absorbance at 490 nm with a microplate spectrophotometer.

### 2.7. FPA-FTIR Microspectroscopy

FT-IR microspectroscopy technique was performed to investigate the effect of the PIT on 3T3-L1 adipocyte cells following the method of Eumkeb et al. and Dunkhuntod et al. [11, 24] with minor modifications. In brief, 3T3-L1 cells were seeded at the density 5×10^5^ cells/well in a 24-well plate. The samples were divided into 4 groups, including differentiated group (DIF), PIT at 750 *μ*g mL^−1^ (PIT 750), Simvastatin at 1.67 *μ*g mL^−1^ (SIM 1.67), and nondifferentiate group (ND, preadipocytes). The 3T3-L1 cells were collected after treatment for 10 days and centrifuged at 400* × g* for 5 min. Cells were washed with 0.85% NaCl and recentrifuged at 400* × g *for 5 min. Cell pellets were dropped onto Barium Fluoride (BaF_2_) optical window 13 mm Ø x 2 mm (Crystran, Crystran Ltd) and air vacuum dried for 30 min in a desiccator to eliminate the excess water. The dropped cell slides were kept in a desiccator until analysis with FTIR.

FTIR spectra were performed by using a spectroscopy facility, at the Synchrotron Light Research Institute (Public Organization), Thailand. FTIR spectra were obtained on a Bruker Vertex 27 spectrometer coupled with a Bruker Hyperion 3000 microscope (Bruker Optics Inc., Ettlin-Gen, Germany). The microscope was equipped with nitrogen-cooled 64x64 element MCT, FPA detector, which allowed simultaneous acquisition of spectral data with a 15 x objective.

The spectra were obtained in the transmission mode with the wavenumber range of 4000-700 cm^−1^. Each of the images used to construct 4x4 binning FTIR image mosaic, 4 cm^−1^ spectral resolution, and 64 scans. The area of the sample, from which single spectra were acquired, was approximately 20 *μ*m x 20 *μ*m. OPUS 7.2 software (Bruker Optics Ltd, Ettlingen, Germany) was used to acquire FTIR spectral data and control instrument system.

The spectra of DIF, PIT (750), SIM (1.67), and ND groups were identified by principal component analysis (PCA) using variability of the Unscrambler 10.1 software (CAMO Software AS, Oslo, Norway). The spectral range of 3000-2800 cm^−1^ and 1800-850 cm^−1^ was used for WEP-treated cells. The preprocessing of the spectra was performed by second derivative transformations using Savitzky-Golay algorithm (nine smoothing points) and normalised with extended multiplicative signal correction (EMSC) using the spectral regions from 3000-2800 cm^−1^ and 1800-950 cm^−1^. This method is used for identifying the overlapping of absorption peaks, reducing variation between replicates spectra, and correcting for baseline shift. Score plots (2D) and loading plots were used to represent the different classes of data and relations among variables of the data set, respectively. The integrated peak areas of the all groups were analysed using OPUS 7.2 software (Bruker).

### 2.8. Pancreatic Lipase Assay

Lipase activity assay was based on the lipase cleaving pNP-laureate to produce a coloured product. The aim of this experiment was to determine if PIT could inhibit lipase activity. Any inhibitory activity was compared to Orlistat, a known inhibitor of lipase activity. The method was based on the method of Guo et al. [2]. In brief, porcine pancreas lipase type 2 was dissolved in distilled water at a concentration of 5 mg mL^−1^. The solution was centrifuged at 10,000* × g* for 5 min, and the supernatant was collected. Reaction substrate was prepared by pNP laurate in reaction buffer (100 mM Tris buffer pH 8.2). 0.1% (w/v) pNP laurate was mixed with 5 mM sodium acetate (pH 5.0) containing 1% Triton X-100 and was heated in boiling water for 2 min until all solid matters were dissolved. After that, the solution was mixed well and cooled to room temperature. All test samples were dissolved in 50% DMSO in reaction buffer. Then 20 *μ*L of the sample and 30 *μ*L of lipase were mixed and added to 40 *μ*L reaction buffer, and the reaction was started by adding 30 *μ*L substrate solution. 50% DMSO instead of the sample was performed as a negative control, and the solution of Orlistat was used as a positive control. Sample blank for each test sample was prepared by the reaction solutions without enzyme. The mixtures were incubated at 37°C for 6 h and measured at 409 nm using a microplate spectrophotometer. The inhibition rate (%) was described as

(1) Inhibitionrate%=1−ODsample−ODsample  blankODnegative  control×100

### 2.9. Determination of Total Phenolic Content (TPC)

The total phenolic content was investigated using the Folin–Ciocalteu assay as previously described by Singleton and Rupasinghe et al. [15, 25]. In brief, 100 *μ*L of 0.2 N of Folin-Cioculteu was pipetted into a 96-well microtitre plate. This was followed by the addition of either 20 *μ*L of the PIT or various concentrations of gallic acid prepared in methanol (0-0.0625 mg mL^−1^). Finally, 80 *μ*L of 7.5% (W/V) sodium carbonate was added and the mixture was incubated at room temperature for 2 hours. The absorbance of the blue colour solution was measured at 765 nm by spectrophotometry, and the total phenolic content was determined using a gallic acid standard curve. The results were expressed as mg gallic acid equivalents (mg GAE/g) per gramme of dry weight.

### 2.10. Determination of Total Flavonoid Content (TFC)

The total flavonoid content was measured using an aluminium chloride colourimetric assay [26, 27]. In brief, 125 *μ*L deionised water was pipetted into a 96-well microtitre plate. This was followed by either the addition of 25 *μ*L standard catechin at various concentrations (0-0.4 mg mL^−1^) or the PIT. Upon completion of the addition of standards or PIT, 10 *μ*L of 5% NaNO_2_ was also added. The mixture was incubated at room temperature for 6 min. To initiate a colour change, 15 *μ*L of 10% AlCl_3_ solution was added. The solution was allowed to stand for 5 min at room temperature. To prevent a further reaction, 50 *μ*L of 1 M NaOH was added and shaken in microplate reader spectrophotometry for 5 min before measuring absorbance at 595 nm. The total flavonoid content was determined using a catechin standard curve. The results were presented as mg catechin equivalents (mg CE/g) per gramme of dry weight.

### 2.11. LC-MS/MS Instrument and Conditions

The chemical characteristic of the PIT was investigated by using LC-MS/MS instrument. The LC-MS/MS system was made up of a combination of chromatographic separation Agilent HPLC 1290 Infinity and the mass analyzer 6490 Triple Quad LC/MS Agilent Technologies equipped with electrospray ionization (ESI) source system, consisting of an autosampler, a binary pump, and vacuum degasser. The chromatographic separation was set on Agilent ZORBAX Rapid Resolution High Definition (RRHD) SB-C18, 2.1 mm id x 150 mm (1.8 *μ*m). Mobile phase system used solvent A and solvent B which consisted of 1% formic acid in water and 1% formic acid in acetonitrile, respectively. Combination of both solvents in LC system was set at a ratio of solvent A: solvent B, 100:0 with gradient elution: from 30% solvent B at 10 min and 100% solvent B at 30 min at a flow rate of 0.2 mL/min. The column temperature was maintained at 25°C, and the sample injection volume was set at 5 *μ*L. The stock solutions of standards (4-CQ, 5-CQ, 3,4-CQ, 3,5-CQ, and 4,5-CQ) were prepared to a final concentration of 1000 *μ*g mL^−1^ by dissolving in methanol, and working solutions were diluted by methanol to obtain the desired concentration and PIT sample working solution was prepared to a final concentration of 1500 *μ*g mL^−1^.

### 2.12. Statistical Analysis

All the data were expressed as a mean ± standard deviation (SD). The statistical significance difference between treatment and control groups of cell viability, the amount of lipid accumulation, biomolecular changes, and lipase activity was analysed by one-way analysis of variance (ANOVA) with a Turkey's HSD post hoc test. Values were considered statistically significant when* p* < 0.05 and data were representative of at least three independent experiments.

## 3. Results

### 3.1. *In Vitro* 3T3-L1 Cytotoxic Test (MTT Assay)

**Figure 1:**
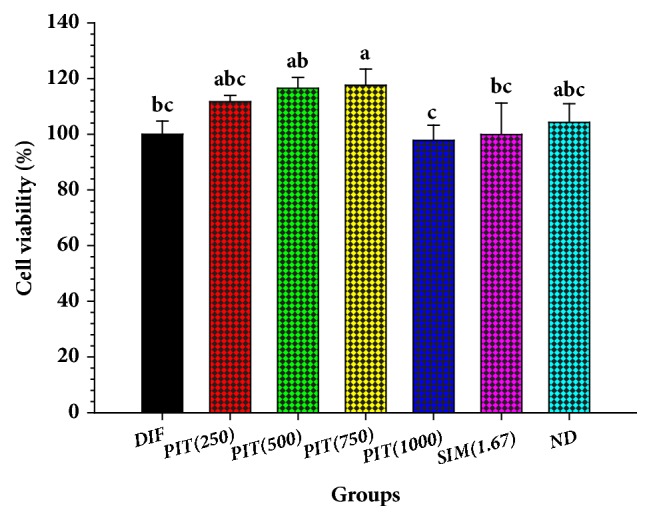
The effect of* Pluchea indica* (L.) tea [15] on the viability of 3T3-L1 preadipocytes. DIF = differentiate 3T3-L1 adipocytes; PIT(250) = PIT at 250 *μ*g mL^−1^ treated-3T3 adipocytes; SIM(4) = Simvastatin at 1.67 *μ*g mL^−1^ treated-3T3 adipocytes; ND = 3T3-L1 preadipocytes (nondifferentiated cells). Means ± SD are illustrated for three replicates. Means with the same superscript are not significantly different from each other (Tukey's HSD test,* p* < 0.05).

Concentrations of the PIT from 250 – 1000 *μ*g mL^−1^ did not significantly affect the viability of 3T3-L1 preadipocytes viability compared to control untreated cells (Figure 1), as assessed by the MTT assay (*p* > 0.05). In all subsequent experiments, doses of 1000 *μ*g mL^−1^ or less were used.

### 3.2. Effect of the PIT on 3T3-L1 Preadipocyte Differentiation and Lipid Accumulation

**Figure 2:**
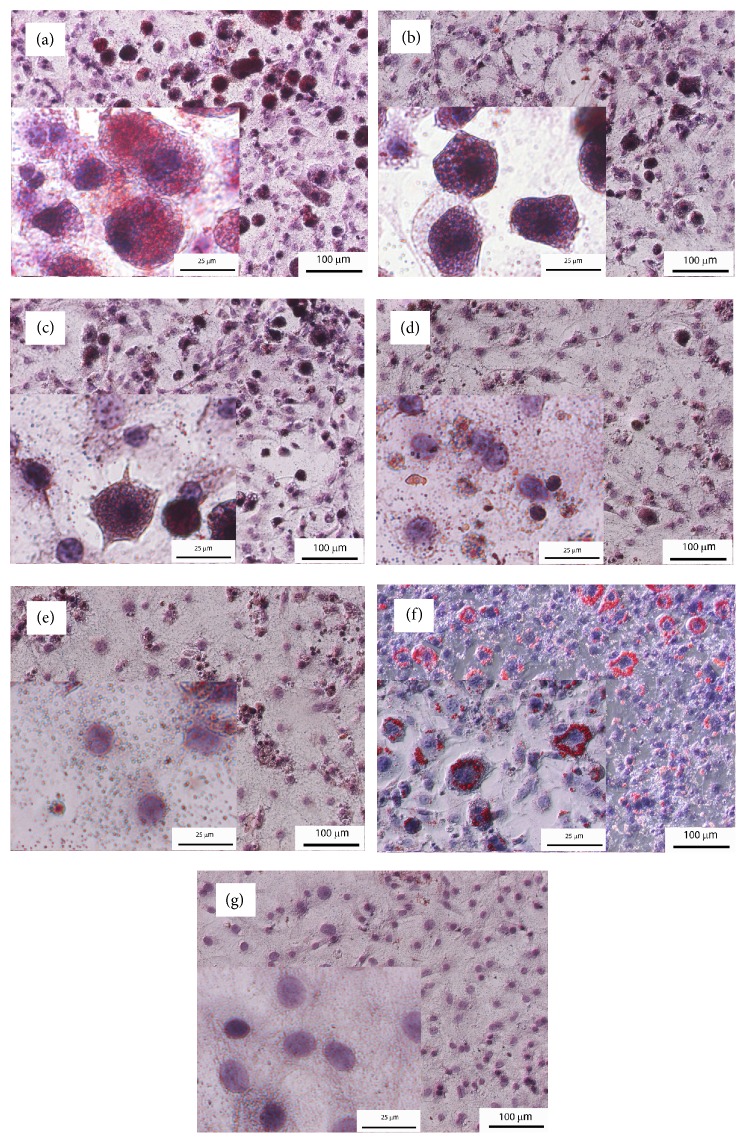
Microscopic imaging of intracellular lipid after Oil Red O and haematoxylin staining of samples.** (a)** = Differentiate 3T3-L1 adipocytes (untreated adipocytes);** (b)** = PIT at 250 *μ*g mL^−1^ treated adipocytes;** (c)** = PIT at 500 *μ*g mL^−1^ treated adipocytes;** (d)** = PIT at 750 *μ*g mL^−1^ treated adipocytes;** (e)** = PIT at 1000 *μ*g mL^−1^ treated adipocytes;** (f)** = Simvastatin at 1.67 *μ*g mL^−1^ treated adipocytes;** (g)** = 3T3-L1 preadipocytes (nondifferentiated cells) (original magnification at x100, scale bar; 100 *μ*m and* Inset* view at x600, scale bar; 25 *μ*m).

**Figure 3:**
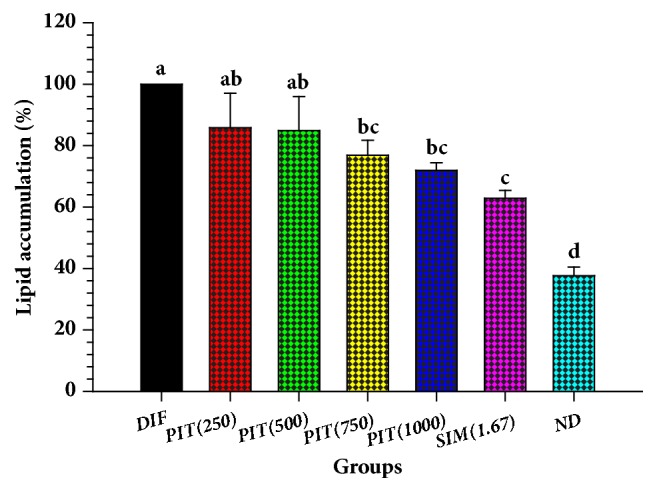
The figure graphically represents the effect of the PIT on the percentage of intracellular lipid in 3T3-L1 differentiated cells after Oil Red O staining. DIF = differentiate 3T3-L1 adipocytes (untreated adipocytes); PIT(250) = PIT at 250 *μ*g mL^−1^ treated adipocytes; SIM(1.67) = Simvastatin at 1.67 *μ*g mL^−1^ treated adipocytes; ND = 3T3-L1 preadipocytes (nondifferentiated cells). Means ± SD are illustrated for three replicates. Means with the same superscript are not significantly different from each other (Tukey's HSD test,* p* < 0.05).

During differentiation of 3T3-L1 preadipocytes to adipocytes, the cells were treated with the PIT at various concentrations (250, 500, 750, and 1000 *μ*g mL^−1^), and the intracellular lipid level was quantified using an Oil Red O staining method. The 3T3-L1 preadipocytes exposure to differentiation medium resulted in a significant increase of lipid accumulation in comparison to untreated adipocytes (DIF) (*p* < 0.05) (Figure 3). Microscopic observation of Oil Red O and hematoxylin-stained cells exhibited that PIT decreased Oil Red O stained droplets of mature adipocytes in a dose-dependent manner (Figure 2). The intracellular lipid accumulation showed that PIT at 750 and 1000 *μ*g mL^−1^ significantly decreased the intracellular lipid accumulation to 76.87 ± 3.99 and 71.93 ± 2.05, respectively, compared to untreated 3T3-L1 adipocytes (DIF) (*p* < 0.05) (Figure 3). The 33 and 50% inhibitory effects (IC_33_ and IC_50_) of the PIT on lipid accumulation were determined to be 1085.5 ± 129.40 and 1841.07 ± 272.60 *μ*g mL^−1^, respectively. In addition, Simvastatin at 1.67 *μ*g mL^−1^ exhibited a 33% lipid accumulation reduction (IC_33_). The effect of Simvastatin is therefore 650 times more effective than a PIT. Accordingly, the PIT concentration at 750 *μ*g mL^−1^ was chosen to study FTIR microspectroscopy.

### 3.3. Biomolecule Changing Detected by FTIR Microspectroscopy

**Figure 4:**
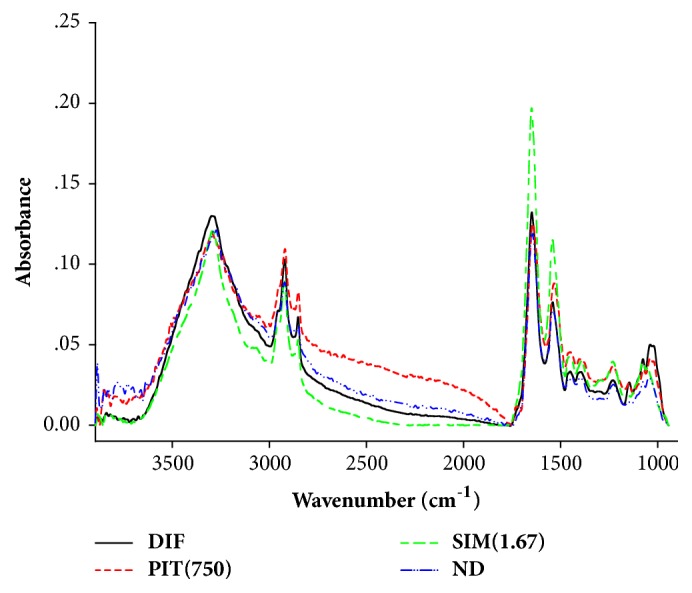
Average original FTIR spectra (4000–950 cm^−1^) obtained from 3T3-L1 cells. DIF = differentiate 3T3-L1 adipocytes (untreated adipocytes); PIT(750) = PIT at 750 *μ*g mL^−1^ treated adipocytes; SIM(1.67) = Simvastatin at 1.67 *μ*g mL^−1^ treated adipocytes; ND = 3T3-L1 preadipocytes (nondifferentiated cells).

**Figure 5:**
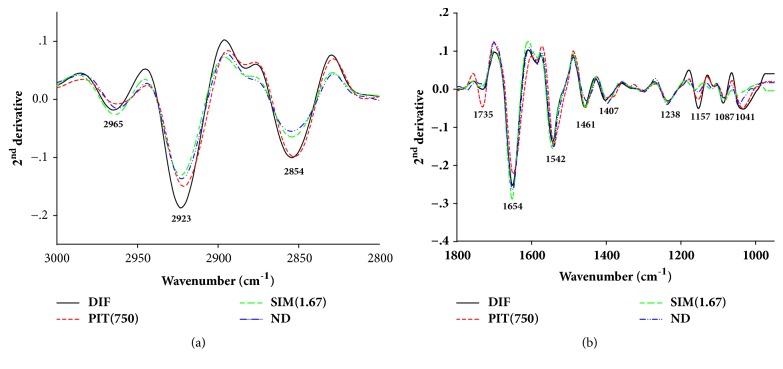
Average the secondary derivative spectra of 3T3-L1 cells. DIF = differentiate 3T3-L1 adipocytes (untreated adipocytes); PIT(750) = PIT at 750 *μ*g mL^−1^ treated adipocytes; SIM(1.67) = Simvastatin at 1.67 *μ*g mL^−1^ treated adipocytes; ND = 3T3-L1 preadipocytes (nondifferentiated cells). The data were represented in two regions:** (a)** lipid regions (3000-2800 cm^−1^) and** (b)** protein, nucleic acid, glycogen, and other carbohydrate regions (1800-950 cm^−1^).

FTIR microspectroscopy was used to determine the biochemical composition of preadipocytes (ND), untreated adipocytes (DIF), Simvastatin- and PIT-treated adipocytes. The FTIR absorption spectrum of the sample at wavelengths between 4000 and 950 cm^−1^ is shown in Figure 4. The three distinct areas of the lipid region (3000-2800 cm^−1^), the protein regions (1700-1500 cm^−1^), carbohydrate, and nucleic acid regions (1300-950 cm^−1^) were investigated. Hence, the spectrum was very difficult to analyse due to the spectral difference between the groups. In order to overcome this problem second derivative of the spectral range at 3000-2800 cm^−1^ and 1800-950 cm^−1^ was analysed (Figures 5(a) and 5(b)).

**Figure 6:**
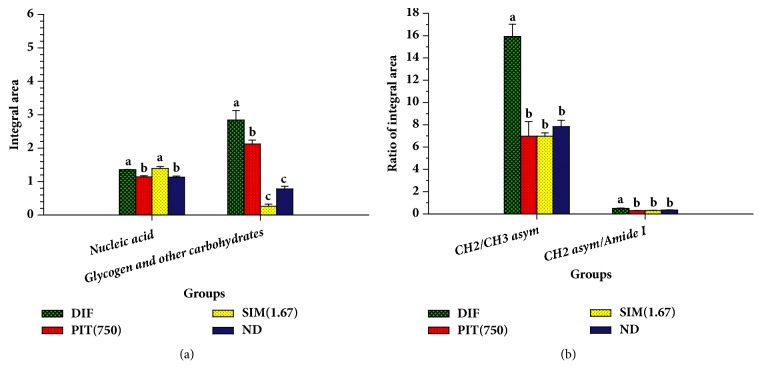
The histogram of integrated areas of 3T3-L1 cells. DIF = differentiate 3T3-L1 adipocytes (untreated adipocytes); PIT(750) = PIT at 750 *μ*g mL^−1^ treated adipocytes; SIM(1.67) = Simvastatin at 1.67 *μ*g mL^−1^ treated adipocytes; ND = 3T3-L1 preadipocytes (nondifferentiated cells).** (a)** The integral area of nucleic acids, glycogen, and other carbohydrates.** (b)** The ratio of the integral area of lipids (CH_2_/CH_3_ asymmetric stretching) and proteins (CH_2_ asymmetric stretching/Amide I). Means ± SD are illustrated for three replicates. Means with the same superscript are not significantly different from each other (Tukey's HSD test,* p* < 0.05).

The strong peak at 2923 cm^−1^ and 2854 cm^−1^ corresponds to the CH_2_ asymmetric and symmetric stretching frequency (mainly lipids, with the little from proteins, carbohydrates, and nucleic acids), respectively [28]. The decrease in signal intensity and area of the peaks (at 2954 cm^−1^ and 2923 cm^−1^), which reflected an absorption peak of lipids of the PIT-treated adipocytes, presented less than untreated adipocytes (Figure 5(a)). Then, we calculated the ratio of the integrated area of several functional groups, including CH_2_ (2938-2906 cm^−1^, centred at 2923 cm^−1^)/CH_3_ (2973-2954 cm^−1^, centred at 2965 cm^−1^) asymmetric stretching that belongs to lipids. The results showed that the ratio of the integrated area of the lipids region in the PIT- and Simvastatin-treated adipocytes displayed significantly less than the untreated adipocytes group (*p* < 0.05) (Figure 6(b)). The major contributor in the spectrum ranges from 1700 to 1500 cm^−1^, which is attributed to an absorption peak of proteins amide I and II. The ratio of integrated area of several functional groups, including CH_2_ asymmetric stretching (2938-2906 cm^−1^, centred at 2923 cm^−1^)/amide I (1674-1624 cm^−1^, centred at 1654 cm^−1^), that belong to proteins of the PIT- and simvastatin-treated adipocytes displayed significantly less integral area ratio than the untreated adipocytes group (*p* < 0.05) (Figure 6(b)).

The signal intensity and area of the peaks of the PIT- and simvastatin-treated adipocytes at 1157 cm^−1^ and 1041 cm^−1^ which are attributed to an absorption peak of C–O vibrations from glycogen and other carbohydrates [29] undoubtedly exhibited significantly less than untreated 3T3-L1 adipocytes (*p* < 0.05) (Figure 6(a)). The functional group of PO_2_ stretching mode from mainly nucleic acids at 1238 cm^−1^ and 1087 cm^−1^ regions of the PIT-treated adipocytes displayed signal intensity and area less than the untreated adipocytes (Figure 5(b)) and demonstrated significantly less integrated area than that of untreated adipocytes group (*p* < 0.05) (Figure 6(a)) [30].

**Figure 7:**
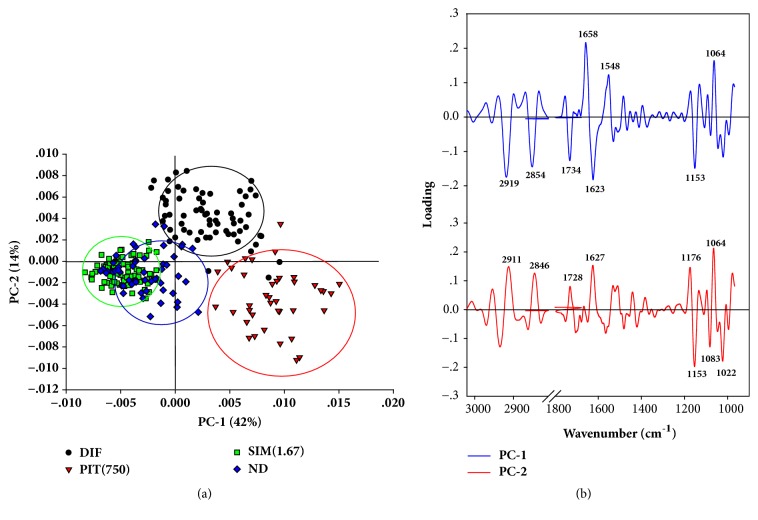
PCA analysis of FTIR spectral ranges 3000-2800 cm^−1^ and 1800-950 cm^−1^ giving PCA score plot (**a**) and PCA loading plot (**b**). The 2D PCA score plots showed the clustering separation spectra between groups. DIF = differentiate 3T3-L1 adipocytes (untreated adipocytes); PIT(750) = PIT at 750 *μ*g mL^−1^ treated adipocytes; SIM(1.67) = Simvastatin at 1.67 *μ*g mL^−1^ treated adipocytes; ND = 3T3-L1 preadipocytes (nondifferentiated cells) at day 10 after differentiation. The biomarker differences over a spectral range of samples are identified by PC1 and PC2 loading plots.

We further investigated the second derivative spectra using the principal component analysis (PCA). The PCA score plot showed that PIT (750) and DIF cluster were isolated from SIM (1.67) and ND cluster with PC-1 (42%) and the cluster of DIF group were separated from PIT (750), SIM (1.67), and the ND group with PC-2 (14%) (Figure 7(a)). The PCA loading plot was used to detect the wavelength of the spectrum that discriminated the clustering (Figure 7(b)). PC-1 is discriminated by negative loading in the C-H stretching region (centred at 2919 cm^−1^ and 2854 cm^−1^), negative loading of C-O vibrations from glycogen and other carbohydrates at 1153 cm^−1^, positive loading of C-O vibrations from glycogen and other carbohydrates at 1064 cm^−1^, protein amide I, and amide II (positive loading centred at 1658 cm^−1^ and 1548 cm^−1^, respectively), and negative loading from C=O stretching vibrations of lipids ester (centred at 1734 cm^−1^). These results suggest that DIF and PIT (750) groups possess higher lipids, glycogen and other carbohydrates, and proteins than SIM (1.67) and ND groups.

The discrimination along PC-2 expressed the separation between DIF group and other groups which demonstrated positive loading in C-H stretching region (centred at 2911 cm^−1^ and 2846 cm^−1^), negative loading of C-O vibrations from glycogen and other carbohydrates at 1153 cm^−1^ and 1022 cm^−1^, positive loading of C-O vibrations from glycogen and other carbohydrates at 1064 cm^−1^, protein amide I (positive loading centred at 1627 cm^−1^), PO_2_-symmetric stretching vibrations of nucleic acid (negative loading centred at 1083 cm^−1^), and positive loading from C=O stretching vibrations of lipids ester (centred at 1728 cm^−1^). These results seem consistent with PC-1 that DIF group expresses higher lipids, glycogen and other carbohydrates, proteins, and nucleic acid than other groups.

### 3.4. Effect of the PIT on Pancreatic Lipase Activity

**Figure 8:**
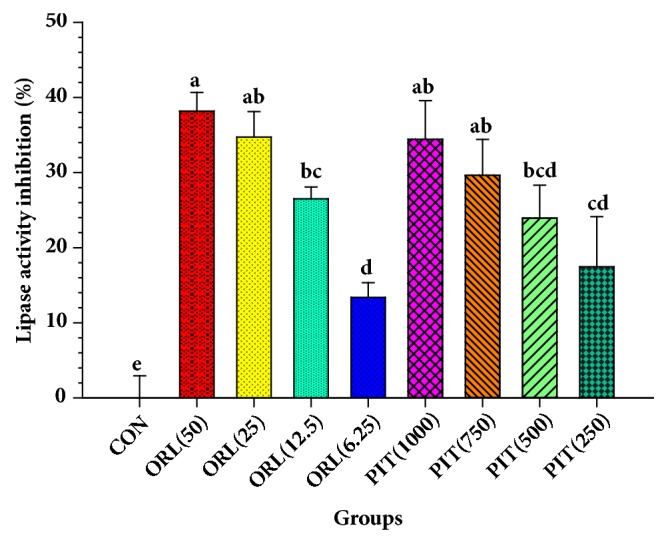
Inhibitory effects of PIT (%) at various concentrations on lipase activity. CON = Control; ORL(12.5) = Orlistat at 12.5 *μ*g mL^−1^; PIT(250) = PIT at 250 *μ*g mL^−1^. Orlistat was used as a positive control. Means ± SD are illustrated for three replicates. Means with the same superscript are not significantly different from each other (Tukey's HSD test,* p *< 0.05).

Pancreatic lipase is an enzyme responsible for the hydrolysis of lipid into free fatty acid and glycerol. The PIT concentrations between 250 and 1000 *μ*g mL^−1^ displayed significantly higher inhibitory lipase activity than those of the controls (*p* < 0.05) (Figure 8). Moreover, the IC_50_ of the PIT for the inhibition of pancreatic lipase was 1708.35 ± 335.85 *μ*g mL^−1^, while the inhibitory effect of the positive control Orlistat at 6.25 to 50 *μ*g mL^−1^ demonstrated an IC_50_ at 68.23 ± 6.67 *μ*g mL^−1^. Under those circumstances, the potential strength of Orlistat on lipase activity inhibition is approximately 25 times greater than the PIT. These results suggest that the inhibitory implications of the PIT on pancreatic lipase activity increased in a dose-dependent manner.

### 3.5. Total Phenolic Content (TPC) of PIT

The total phenolic content was investigated by using the Folin–Ciocalteu colourimetric assay. Gallic acid was used as a standard of the phenolic compound. A standard calibration curve of gallic acid had an R^2^ value of 0.9991 and standard equation y = 51.696x + 0.0624. The total phenolic content was calculated and showed as gallic acid equivalents (GAE)/g of dry weight. The results indicated that total phenolic of the PIT was 107.95 ± 4.87 mg GAE/g of dry weight.

### 3.6. Total Flavonoid Content (TFC) of PIT

Total flavonoid content was investigated by using the aluminium chloride colourimetric assay and expressed regarding catechin equivalent (CE)/g of dry weight. The total flavonoid content was calculated by standard calibration curve of catechin with an R^2^ value of 0.994 and the standard equation of y = 3.3315x + 0.0825. The results showed that the total flavonoid content of PIT was 95.33 ± 0.48 mg CE/g of dry weight.

### 3.7. Chemical Identification of PIT by LC-MS/MS

**Figure 9:**
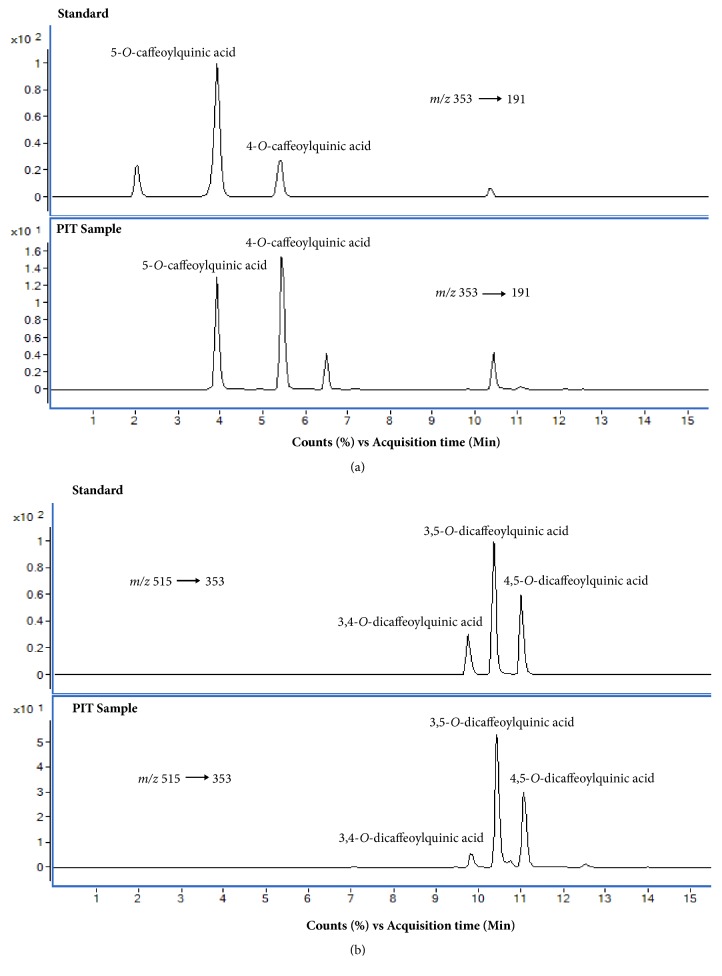
MRM chromatograms of PIT and standards (4-CQ, 5-CQ, 3,4-CQ, 3,5-CQ and 4,5-CQ) of MRM transition at m/z 353→191.0** (a)** and at m/z 515→353** (b)**.

Negative ion mode was selected for ESI-MS analysis in this study, and the Multiple Reaction Monitoring (MRM) mode has been used for identification. Two pairs of MRM transition were selected at *m*/*z* 353.1→191.0 and 515→353. MRM chromatograms of PIT and standards (4-CQ, 5-CQ, 3,4-CQ, 3,5-CQ, and 4,5-CQ) of MRM transition at *m*/*z* 353→191.0 are shown in Figure 9(a). The result showed that PIT had been detected 4-CQ and 5-CQ in the extract.  Figure 9(b) revealed the chromatograms of PIT and standards of MRM transition at* m/z* 515→353 which explained that 3,4-CQ, 3,5-CQ, and 4,5-CQ were the main ingredients of the PIT.

The quantification of the analytes was performed. We focused on the highest peak of chromatogram that was 3,5-CQ, and the result from MRM data acquisitions showed that PIT concentration 1500 *μ*g mL^−1^ composed of 3,5-CQ 169.93 *μ*g mL^−1^.

## 4. Discussion

Obesity and hyperlipidemia are caused by an imbalance between energy intake and energy expenditure which can cause some of the health problems such as type 2 diabetes, ischemic heart disease, stroke, and cancer [2, 31]. Obesity and hyperlipidemia can be treated with conventional medicine example Simvastatin for dyslipidemia and Orlistat for obesity, but the medication has limited efficacies and has some side effects such as rhabdomyolysis [32], nausea, and vomiting [33]. Therefore, traditional medicine is an essential alternative medicine to alleviate these diseases and may have minor side effects when compared with modern medicine. Moreover, if traditional medicine can be used alone or combined with modern medicine, it will decrease the use of modern medicine or reduce the dose and side effects of modern medicine as well [34, 35].

*P. indica* (L.) was reported that it had many therapeutic indications such as decreasing blood sugar [19] and reducing blood pressure. In addition, folk medicine can help lower the hyperlipidemia, but this indication has not proven the efficacy by the researcher. Nevertheless,* P. indica* (L.) has the potential for prevention and treatment of metabolic syndrome because it may have effects on both lower blood glucose and blood lipid. Moreover, no work has been done on its efficacy and safety.

The inhibition of adipogenesis using 3T3-L1 cells can predict the efficacy of* P. indica* (L.) on lipid formation inhibition [23]. In addition, the use of MTT assay that has been widely used to evaluate cell viability can investigate the preliminary safety and acute toxicity of the compound. The pancreatic lipase activity investigation can suggest the efficacy of PIT on lipid absorption inhibition from the gastrointestinal tract to the blood results in blood lipid reduction [2].

The MTT assay can detect the cytotoxicity of the compound on the cells. Viable cells can generate MTT to formazan which has a purple colour. The MTT results showed that the viability of cells treated with a PIT at 250–1000 *μ*g mL^−1^ was not significantly different compared to the controls (DIF) (*p* > 0.05). These findings provide evidence that PIT at ≤ 1000 *μ*g mL^−1^ is safe for 3T3-L1 cells. Our results are in substantial agreement with those of Srisook et al. and Pramanik et al. that* P. indica* (L.) alone at 25 to 400 *μ*g mL^−1^ showed no cytotoxic effect in RAW 264.7 macrophage cells [36] and at 400 mg kg^−1^ did not cause any side effects to rat [19].

The investigation of the adipogenesis of 3T3-L1 cell can be tested by using Oil Red O staining technique and illuminated by a microscope. The microscopic observation results of Oil Red O and hematoxylin-stained cells displayed that the PIT decreased Oil Red O stained droplets of mature adipocytes in a dose-dependent manner. Also, we can detect the quantity of lipid by eluting Oil Red O dye by 100% Isopropanol and measuring lipid accumulation by microplate spectrophotometer. Interestingly, the effect of the PIT at 750 and 1000 *μ*g mL^−1^ did not show significantly different in lipid accumulation compared to 1.67 *μ*g mL^−1^ of Simvastatin, which is a leading lipid-lowering drug.

The main active ingredients of the PIT have been investigated by using LC-MS/MS technique. The result showed that the main chemical compositions of the PIT were caffeoylquinic acid derivatives which correlated with Kongkiatpaiboon et al. [37]. Interestingly, caffeoylquinic acid has been reported for its effect on hyperlipidemia, where the enriching of caffeoylquinic acid derivatives in* Pandanus tectorius *fruit extract moderated hyperlipidemia and improved the liver lipid profile in hamsters fed high fat diet and these effects may be caused by increasing the expression of* PPARα* and its downstream genes and by upregulation of LPL and* AMPK* activities [38].

The total phenolic and total flavonoid content results are in substantial agreement with those of Susetyarini et al. [39] that tannin which is phenolic compound was found in* P. indica* (L.). Furthermore, these results are consistent with Andarwulan et al. that the active flavonoid and phenolic compounds were discovered in* P. indica* (L.) [40]. Likewise,* P. indica* (L.) appeared to possess eudesmane derivatives, terpene glycosides, benzenoids, phenylpropanoids, lignan glycosides, stigmasterol glucoside, quercetin, and chlorogenic acid [36].

Furthermore, our data are consistent with those of Hsu et al. that flavonoids and phenolic acids caused 3T3-L1 cell cycle arrest in the G1 phase that may play a role in the control adipogenesis of 3T3-L1 cell and might have the further implication of* in vivo* antiobesity effects [41]. Apart from this, the previous study reported that quercetin, which is the one of active ingredients in* P. indica* (L.) [37], could inhibit adipogenesis in 3T3-L1 adipocyte cell [42]. Moreover, quercetin was reported to have the ability to improve hypertriglyceridemia, alleviate hypercholesterolemia, and elevate HDL-cholesterol in db/db mice by decreasing the expression of peroxisome proliferator-activated receptor-*α* (*PPAR-α*) and sterol regulatory element-binding protein-1c (*SREBP-1c*) and reducing acetyl-CoA carboxylase (ACC) activity [43]. These results provide evidence that PIT may decrease the adipogenesis of the cells and have the potential to develop to be a herbal supplement that can prevent hyperlipidemia after efficacy and toxicity in animal and human have been investigated.

FTIR microspectroscopy has previously been used to characterise the spectral properties of biological change in various samples [11, 44]. These results showed that the integrated areas of lipids, proteins, nucleic acids, glycogen, and carbohydrates of the PIT-treated adipocytes group were significantly lower than untreated adipocytes group (DIF) (*p* < 0.05) (Figures 6(a) and 6(b)). Our results are in correspondence with those of Dunkhuntod et al. that baicalein reduces lipids, proteins, glycogen, and other carbohydrates in baicalein-treated 3T3-L1 adipocytes compared to untreated 3T3-L1adipocytes [11]. Moreover, the decreasing of integrated area of lipids of FTIR is consistent with the Oil Red O staining results. PCA analysis exhibited discrimination of four clusters of the FTIR spectra of preadipocyte (ND), untreated adipocyte (DIF), and Simvastatin- and PIT-treated adipocytes (Figures 7(a) and 7(b)).

The lipase is the enzyme responsible for the digestion of the lipid before it is absorbed into the bloodstream. The inhibition of the enzyme can reduce the absorption of lipid which can minimise the risk of obesity and hyperlipidemia disease. The results indicated that PIT could inhibit the enzyme lipase activity in a dose-dependent manner and had potency less than Orlistat for 25.04 times. These results are in correspondence with with those of Zhang et al. and Birari that polyphenolic compound like flavonoid could inhibit the enzyme lipase activity and reduce lipid absorption in the intestine [7, 45]. Moreover, Dunkhunthod et al. reported that baicalein could inhibit pancreatic lipase activity [11]. Also, the polyphenol-rich plants extract derived from grape seed extract, fermented oats, berry, or strawberry had been reported to inhibit lipases activity, and TPC enhanced the pancreatic lipase inhibitory effect [46, 47]. Thus, the inhibitory effect of the PIT may depend on the amount of TPC and TFC, which vary with the PIT concentrations.

## 5. Conclusions

Our results provide evidence that the PIT can decrease lipid accumulation in 3T3-L1 adipocytes and primarily inhibit adipogenesis. The PIT also modifies the lipid, carbohydrate, protein, nucleic acid and glycogen concentrations within the cells. Furthermore, the PIT could inhibit lipase activity* in vitro*. This study also demonstrates that FTIR microspectroscopy can provide valuable information on the biochemistry of 3T3-L1 adipocytes. The safety of PIT on 3T3-L1 adipocytes suggests that PIT may be developed to hyperlipidemia or anti-obesity herbal. However, these* in vitro* results have to be still confirmed in an animal or human test to achieve blood and tissue therapeutic levels.

## Data Availability

The datasets used and analysed during the current study are available from the corresponding author on reasonable request.
